# Behavior of *Salmonella* and *Listeria monocytogenes* in Raw Yellowfin Tuna during Cold Storage

**DOI:** 10.3390/foods5010016

**Published:** 2016-03-02

**Authors:** Chengchu Liu, Jing Mou, Yi-Cheng Su

**Affiliations:** 1 Sea Grant College Extension Program, University of Maryland, Princess Anne, MD 21853, USA; cathyliu@umd.edu; 2 Seafood Research and Education Center, Oregon State University, Astoria, OR 97103, USA; moujing.christy@gmail.com

**Keywords:** survival, foodborne pathogens, *Salmonella*, *Listeria monocytogenes*, yellowfin tuna, refrigeration and frozen storage

## Abstract

Behavior of *Salmonella* and *Listeria monocytogenes* in raw yellowfin tuna during refrigeration and frozen storage were studied. Growth of *Salmonella* was inhibited in tuna during refrigerated storage, while *L. monocytogenes* was able to multiply significantly during refrigerated storage. Populations of *Salmonella* in tuna were reduced by 1 to 2 log after 12 days of storage at 5–7 °C, regardless levels of contamination. However, populations of *L. monocytogenes* Scott A, M0507, and SFL0404 in inoculated tuna (10^4^–10^5^ CFU/g) increased by 3.31, 3.56, and 3.98 log CFU/g, respectively, after 12 days of storage at 5–7 °C. Similar increases of *L. monocytogenes* cells were observed in tuna meat with a lower inoculation level (10^2^–10^3^ CFU/g). Populations of *Salmonella* and *L. monocytogenes* declined gradually in tuna samples over 84 days (12 weeks) of frozen storage at −18 °C with *Salmonella* Newport 6962 being decreased to undetectable level (<10 CFU/g) from an initial level of 10^3^ log CFU/g after 42 days of frozen storage. These results demonstrate that tuna meat intended for raw consumption must be handled properly from farm to table to reduce the risks of foodborne illness caused by *Salmonella* and *L. monocytogenes*.

## 1. Introduction

*Salmonella* and *Listeria monocytogenes* are two leading human pathogens responsible for foodborne hospitalization and death in the United States. According to the Foodborne Outbreak Online Database (FOOD Tool) data of Centers for Diseases Control and Prevention (CDC), a total of 18,211 foodborne disease outbreaks with 358,391 illness, 13,715 hospitalization, and 318 death occurred from 1998 to 2014 in the United States [1]. Among all known foodborne pathogens, *Salmonella* was linked to 2273 outbreaks (12.5% of total foodborne disease outbreaks), 61,630 illness (17% of total foodborne illness), 6952 hospitalization (50.1% of total foodborne hospitalization), and 79 death (24.8% of total foodborne death); while *L. monocytogenes* was responsible for 58 outbreaks (0.3% of total foodborne disease outbreaks), 766 illness (0.2% of total foodborne illness), 521 hospitalization (3.8% of total foodborne hospitalization), and 116 death (39.6% of total foodborne death). Together, *Salmonella* and *L. monocytogenes* attributed to 7473 hospitalization (53.9% of total foodborne hospitalization), and 195 death (61.3% of total foodborne death) in the U.S. during 1998–2014.

Seafood is a major global food commodity and an important part of a healthy diet. As with any type of food, seafood consumption is not risk-free. In fact, seafood is one of the four food categories with the highest risk responsible for large numbers of foodborne illnesses and outbreaks in U.S. during the past decade [2]. Seafood products have been shown to be often contaminated with *Salmonella* [3,4] and *L. monocytogenes* [5,6,7,8]. A study conducted by field laboratories the U.S. Food and Drug Administration (FDA) demonstrated the presence of *Salmonella* in a variety of fish and shellfish, including ready-to-eat (RTE) seafood products with an overall incidence of *Salmonella* in 1.3% of domestic and 7.2% of import seafood. Among all test samples, the incidence of *Salmonella* in ready-to-eat (RTE) seafood intended for raw consumption was 0.47% for domestic and 2.6% for import products [9]. As for *L. monocytogenes*, a survey of minced tuna collected from retail stores in Japan between 2002 and 2003 revealed that *L. monocytogenes* was present in 14.3% of the raw material [10]. In addition, the incidence of *L. monocytogenes* in RTE minced tuna, fish roe, and smoked fish was 5.7%, 12.1% and 25% [7,8], respectively.

Since ready-to-eat (RTE) food are normally consumed without cooking, RTE seafood products (sashimi, sushi, smoked fish, seafood salads or dips) represent a high risk for causing foodborne illness if they are contaminated with foodborne pathogens and not handled properly during storage, preparation and serving. *Salmonella* and *L. monocytogenes* in RTE seafood products are significant food safety concerns. In 2012, a large outbreak of *Salmonella* infection associated with consumption of sushi containing imported frozen raw yellowfin tuna occurred in the United States. A total of 425 persons from 28 states and the District of Columbia were infected by *Salmonella* Bareilly (410 cases) and *Salmonella* Nchanga (15 cases) with 55 victims being hospitalized [11]. In 2015, another outbreak of salmonellosis was liked to tuna sushi with a total of 65 people infected with Salmonella Paratyphi (64 people) and Salmonella Weltevreden (1 person) in 11 states, including Arizona (12), California (35), Illinois (1), Michigan (2), Minnesota (4), Mississippi (1), New Mexico (6), South Dakota (1), Virginia (1), Washington (1), and Wisconsin (1) [12]. In addition, a *Salmonella* Thompson outbreak (866 cases) associated with consumption of cold-smoked salmon was reported in the Netherlands in 2012 [13]. Although the incidence of foodborne listeriosis was relatively low compared with other foodborne pathogens, foodborne listeriosis outbreaks have occurred in the U.S., Japan, New Zealand, Germany, England, France, and other countries over the past 2 decades [8]. During the period of August 1994 to June 1995, a cluster of listeriosis cases (nine patients with two deaths) linked to the consumption of RTE rainbow trout product was reported in Sweden [14]. An outbreak of five cases of febrile gastroenteritis in Finland was reported associated with consumption of vacuum-packed, cold-smoked rainbow trout that was contaminated with *L. monocytogenes* [15].

Refrigeration and freezing are the most common means used to ensure seafood safety and quality. However, the efficiencies of these processes on inhibiting or retarding the growth of certain foodborne pathogens in seafood products haven’t been well documented yet. No information is available on survival of *Salmonella* and *L. monocytogenes* in raw tuna during refrigerated and frozen storage. The outbreaks described above indicate that *Salmonella* and *L. monocytogenes* carried by raw or ready-to-eat seafood, such as raw tuna sushi and smoked salmon, has the ability to survive at refrigeration and freezing temperatures and cause human infection when the product is consumed. The objective of this study is to investigate the behavior of *Salmonella* and *L. monocytogenes* in raw yellowfin tuna during refrigerated and frozen storage.

## 2. Materials and Methods

### 2.1. Target Pathogen Strains and Culture Preparation

Two strains of *Salmonella* (*S*. Weltevreden SFL 0319 isolated from shrimp and *S*. Newport ATCC 6962 isolated from meat) and three stains of *L*. *monocytogenes* (Scott A from clinic samples, and M0507 and SFL0404 both from shrimp samples) were used in this study. Each strain was grown in 10 mL of tryptic soy broth (TSB; BD Bacto™, Becton, Dickinson and Company, Sparks, MD, USA) at 35 ± 2 °C for 10–12 h. One loopful (~ 1 μL) of each enrichment was streaked onto a tryptic soy agar (TSA; BD BBL™ TSA II, Becton, Dickinson and Company) plate and incubated at 35 ± 2 °C overnight (~ 18 h). A single colony on the TSA plate was transferred to 10 mL of TSB and incubated overnight at 35 ± 2 °C to produce a culture suspension of 10^8–9^ CFU/mL. The culture was diluted with Butterfield’s phosphate diluent (BPD, pH 7.2) to 10^5–7^ or 10^4–6^ CFU/mL for high-level or low-level inoculation of samples.

### 2.2. Tuna Samples and Inoculation of Target Pathogens

Frozen raw yellowfin tuna blocks (approximately 450 grams per block) without blood and skin were purchased from local retail stores and stored at −70 °C before use. Initial tests of samples found no *Salmonella* or *Listeria monocytogenes* in the samples.

Frozen tuna samples were thawed in a refrigerator (5–7 °C) overnight and then cut into small cubes (approx. 1.0 cm × 1.0 cm × 1.0 cm). Cut tuna cubes were placed in a sterile container on ice and mixed with each culture suspension thoroughly to achieve a contamination level of 10^3^–10^5^ CFU/g or 10^2^–10^4^ CFU/g. Inoculated samples were aseptically transferred to sterile stomacher bags (15.2 cm × 22.9 cm, Whirl-Pak, Nasco, Modesto, CA, USA) with each bag containing 25 g of sample. All bags were sealed and stored in a refrigerator (5–7 °C) for 14 days or in a walk-in freezer (−18 ± 2 °C) for 12 weeks. Two batches (14 bags/batch) of samples without pathogen inoculation were prepared as controls for determination of aerobic plate counts and psychrotrophic bacterial counts during refrigerated and frozen storage. For refrigeration storage, samples were analyzed for *Salmonella* and *L. monocytogenes* every two days. For frozen study, samples were analyzed every two weeks for 12 weeks.

### 2.3. Determination of Aerobic Plate Counts (APC) and Psychrotrophic Bacterial Counts (PBC)

Pour plate method using trypticase soy agar (TSA) was used to determine aerobic and psychrotrophic bacteria of yellowfin tuna cubes during refrigerated and frozen storage. At each sampling time, two bags of tuna cubes were withdrawn from refrigerator or freezer. The frozen tuna samples were thawed at refrigeration temperature for 2 h before analysis. Each sample was homogenized with 225 mL BPD at speed of 260 rpm for 1 min in a stomacher laboratory blender (Model 400 C, Seward Laboratory Blender Stomacher, Worthing, UK) to prepare a sample suspension (1:10). Serial ten-fold dilutions of the suspension were prepared with the BPD. One mL of each sample dilution was transferred to two petri dishes and mixed with melted TSA (47.5 °C), individually. Solidified TSA plates were inverted and incubated at 35 ± 2 °C for 48 h for aerobic plate counts (APC) and at 7 °C for 10 days for psychrotrophic bacterial counts (PBC) [16]. Results were reported as means (CFU/g) of four determinations.

### 2.4. Enumeration of Target Pathogens in Inoculated Tuna

Surface-plating method on selective media specific for *Salmonella* (xylose lysine deoxycholate agar (XLD), EMD, Darmstadt, Germany) and *L. monocytogenes* (Oxford agar base (BD Difco™, Becton, Dickinson and Company, Sparks, MD, USA) supplied with Oxford agar supplement (HiMedia, HiMedia Laboratories Pvt. Ltd., Mumbai, India)) were used to determine the populations of target pathogens in inoculated tuna samples during storage. At each sampling time, two bags of tuna samples were removed from refrigerator or freezer to prepare sample suspension as previously described. Each sample dilution (0.1 mL) was spread on XLD or Oxford agar plates in duplicate plates with a sterile, L-shaped, polypropylene spreader. The XLD and Oxford agar plates were incubated at 35 ± 2 °C for 24 and 24–48 h, respectively. After enumeration, one typical colony (big black colony with opaque pink edge on XLD media for *Salmonella* or black colony surrounded by black halo on Oxford agar for *L. monocytogenes*) was transferred to 10 mL of TSB and incubated overnight (~ 18h) at 35 ± 2 °C for confirmation as *Salmonella* or *L. monocytogenes* by the polymerase chain reaction (PCR) assays using the primers (Table 1) and procedures described by Rahn *et al.* [17] and Chen and Knabel [18], respectively.

### 2.5. Data Analysis

Bacterial counts obtained at different stages of storage were converted to log values and analyzed with One-Way ANOVA and Tukey’s Honestly Significant Difference Test (SAS Version 9.2, SAS Institute, Inc., Cary, NC, USA). Significant differences among means of each treatment over time were established at *p* < 0.05.

## 3. Results and Discussion

### 3.1. Changes of Aerobic Plate Counts (APC) and Psychrotrophic Bacterial Counts (PBC) in Tuna during Refrigerated and Frozen Storage

For tuna samples stored at 5–7 °C, the initial levels of APC (3.69 log CFU/g) and PBC (3.98 log CFU/g) of the samples (on day 0) remained unchanged nearly two days and then increased rapidly to 7.36 and 7.61 log CFU/g on day 6, respectively (Table 2).

It is well-known that psychrotrophic bacteria grow better than mesophilic bacteria in seafood during refrigeration storage and cause spoilage of seafood products [19]. Therefore, analysis of psychrotrophic bacteria in seafood is highly recommended due to wide usage of refrigerated storage for seafood [20]. According to the International Commission on Microbiological Specifications for Foods, the microbiological limit for defective quality is 10^7^ CFU/g [21]. Therefore, quality of tuna samples became unacceptable after 6 days of storage at 5–7 °C with a shelf life of less than 6 days. The increases of APC and PBC in tuna samples during refrigerated storage suggested that raw tuna shouldn’t be consumed after being stored in a refrigerator for more than 5 days due to deterioration of products.

For tuna samples stored in a freezer (−18 ± 2 °C), no significant changes in APC or PBC were observed after 12 weeks of frozen storage (Table 3). This indicates that most of bacteria in tuna samples were able to survive during frozen storage at −18 ± 2 °C for nearly 3 months. Therefore, thawed frozen tuna meat should be stored at low temperature (5–7 °C) to retard growth of PBC before consumption.

### 3.2. Behavior of Salmonella and Listeria monocytogenes in Tuna during Refrigeration Storage

**Figure 1:**
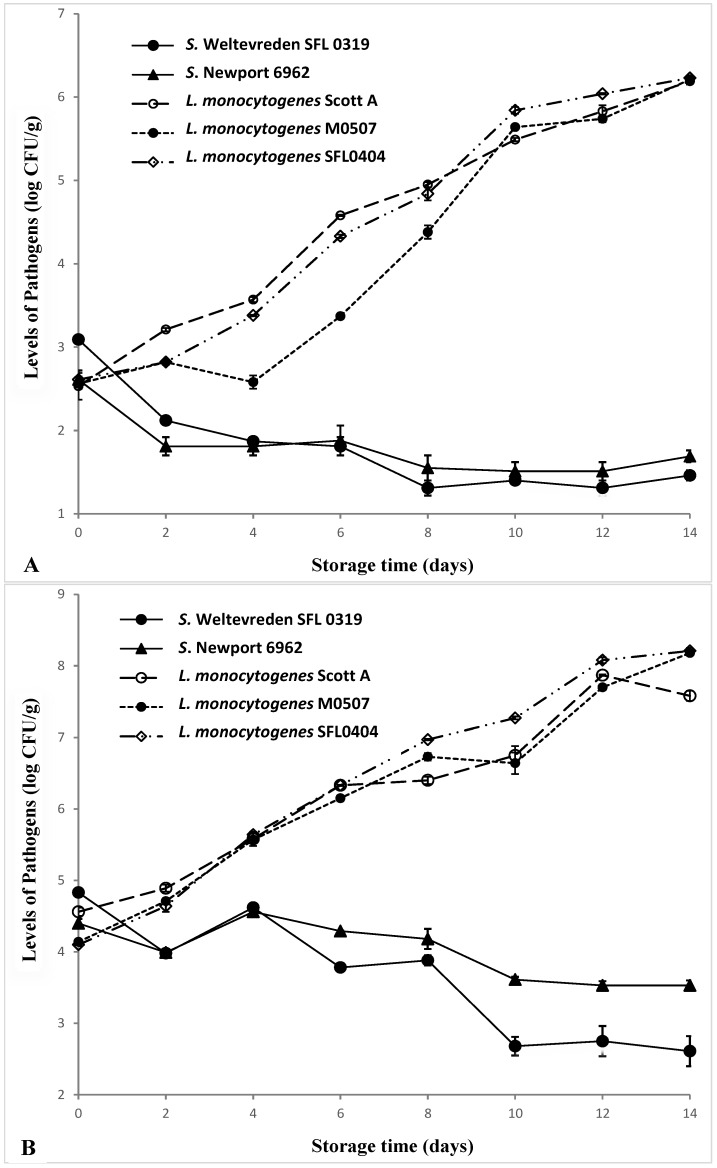
Populations of *Salmonella* and *L. monocytogenes* in inoculated tuna samples during storage at 5–7 °C (**A**: Inoculation level of 10^2–3^ CFU/g; **B**: Inoculation level of 10^4–5^ CFU/g).

*L. monocytogenes* increased remarkably in tuna samples during refrigeration storage at 5–7 °C (Figure 1). All three strains of *L. monocytogenes* in the tuna samples achieved 3–4 log increases after 14 days of storage regardless of inoculation levels. On the other hand, populations of *Salmonella* declined gradually during the refrigeration storage. Among the two *Salmonella* strains used in this study, *S.* Weltevreden SFL 0319 had less cold tolerance than *S.* Newport 6962. Populations of *S.* Weltevreden SFL 0319 in tuna samples declined by 1.63 and 2.22 log CFU/g at inoculation levels of 10^2–3^ and 10^3–4^ CFU/g, respectively. As for *S.* Newport 6962, only 0.8–0.9 log reductions were observed after 14 days of storage at both inoculation levels.

It is well-known that *L. monocytogenes* is a psychrotrophic bacterium able to multiply at temperatures as low as −1°C [22] and grow significantly in a variety of refrigerated food, such as beef strips [23], vegetables [24], and seafood salad [25]. Hudson and Mott [26] investigated growth of *L. monocytogenes* in cold-smoked salmon stored aerobically at 5 °C and reported that the *L. monocytogenes* counts increased by 4–5 log units over 27 days storage period. Another study observed that *L. monocytogenes* levels in seafood salad increased from 1.5 to 7.0 log CFU/g after 22 days of storage at 4 °C [23], even though the pH (4.0–5.1) of seafood salad is not optimal for growth of *L. monocytogenes* [22]. This study demonstrated that *L. monocytogenes* could multiply significantly under aerobic condition at refrigeration temperature and increased by 3–4 log CFU/g in raw yellowfin tuna after 14 days of storage at 5–7 °C.

### 3.3. Survival of Salmonella and L. monocytogenes in Tuna during Frozen Storage

**Figure 2:**
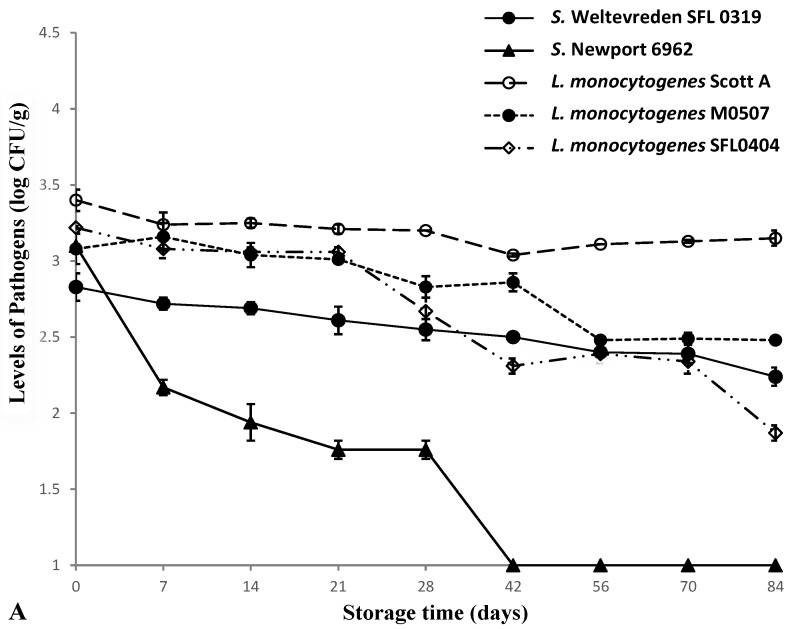

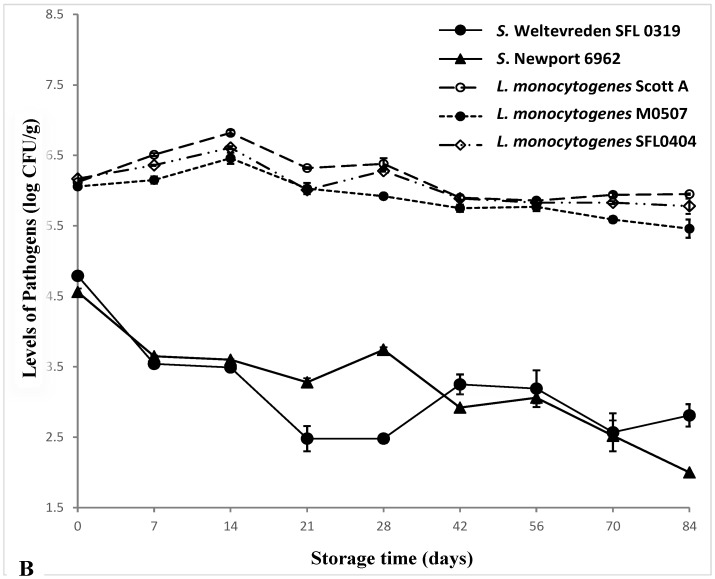
Survival of *Salmonella* and *L. monocytogenes* in inoculated tuna samples during frozen storage at −18 °C (**A**: Inoculation level of 10^3–4^ CFU/g; **B**: Inoculation level of 10^4–6^ CFU/g).

*Salmonella* and *L. monocytogenes* in tuna samples survived during frozen storage even though both pathogens decreased gradually over the 12 weeks of storage regardless of inoculation levels (Figure 2). Compare with *L. monocytogenes*, *Salmonella* was more sensitive to freezing treatment with reductions of 1.98 and 2.56 log CFU/g being observed for *S.* Weltevreden and *S.* Newport, respectively, in samples with an inoculation level of 10^5–6^ CFU/g after 12 weeks. In samples with lower inoculation level (~ 10^3^ log CFU/g), populations of *S.* Weltevreden were reduced from 2.83 to 2.24 log CFU/g after 12 weeks while numbers of *S.* Newport declined from 3.10 log CFU/g to non-detectable level (<10 CFU/g) after 7 weeks of storage.

It has been reported that *Salmonella* in Pacific oysters declined by 2-log after 14 days of storage at −34 °C [27] and *Salmonella* was able to survive up to 9 months in shrimp under −20 °C frozen storage [28]. All these studies clearly indicate that *Salmonella* can survive in frozen products for a period of time regardless the temperature used for frozen storage.

*L. monocytogenes* survived better than *Salmonella* during frozen storage. Generally speaking, less than 1-log unit of reductions of *L. monocytogenes* cells were observed in tuna samples after 12 weeks of storage at −18 °C regardless of inoculation levels. A similar study reported that levels of *L. monocytogenes* were reduced by less than 1-log in frozen fish and shrimp during three months of storage at −20 °C [29]. Another study investigating survival of *L. monocytogenes* reported a 3.69-log reduction in frozen salmon inoculated with a high level (10^8^ CFU/g) and stored at −20 °C for 12 months [30]. These results indicate that frozen products may carry *L. monocytogenes* even after months of frozen storage if the products were contaminated with *L. monocytogenes*.

## 4. Conclusions

Thawed raw tuna became spoiled (aerobic plate counts >10^7^ CFU/g) after 6 days of storage at 5–7 °C. Growth of *Salmonella* was inhibited in tuna during refrigerated storage, while *L. monocytogenes* was able to multiply significantly during refrigerated storage. Even though the populations of *Salmonella* and *L. monocytogenes* declined gradually over 12 weeks of frozen storage at −18 °C, all strains survived frozen storage, except that *Salmonella* Newport 6962 decreased to undetectable level after 7 weeks of frozen storage at inoculation level of 10^3^ log CFU/g. These results demonstrate that raw tuna and other seafood destined for direct consumption as RTE seafood must be handled properly during the whole food chain (from farm to table) to reduce the risks of foodborne illness caused by *Salmonella*, *L. monocytogenes* and other pathogens.

## Figures and Tables

**Table 1:** Primers used in PCR assays for detecting *Salmonella* and *L. monocytogenes*.

Pathogen	Gene	Primer	Sequence (5′–3′)	Size (bp)
*Salmonella*	*inv*A	*inv*AF	GTGAAATTATCGCCACGTTCGGGCAA	284
		*inv*AR	TCATCGCACCGTCAAAGGAACC	
*L. monocytogenes*	*lmo*2234	*lmo*F	TGTCCAGTTCCATTTTTAACT	420
		*lmo*R	TTGTTGTTCTGCTGTACGA	

**Table 2:** Aerobic plate counts (APC) and psychrotrophic bacterial counts (PBC) of tuna samples stored at 5–7 °C.

Bacterial Counts	Day 0	Day 2	Day 4	Day 6	Day 8	Day 10	Day 12
APC	3.69 ± 0.06 ^a^	3.80 ± 0.09 ^a^	4.95 ± 0.05 ^b^	7.36 ± 0.02 ^c^	8.03 ± 0.08 ^d^	8.53 ± 0.17 ^e^	8.75 ± 0.08 ^e^
PBC	3.98 ± 0.02 ^a^	3.98 ± 0.06 ^a^	5.57 ± 0.14 ^b^	7.61 ± 0.01 ^c^	8.73 ± 0.05 ^d^	9.11 ± 0.06 ^e^	9.18 ± 0.01 ^e^

Bacterial counts (Log CFU/g) were reported as means of four determinations ± standard deviation. Data with a different letter in the same row are significantly different (*p* < 0.05).

**Table 3:** Aerobic plate counts (APC) and psychrotrophic bacterial counts (PBC) of tuna samples stored at −18 ± 2 °C.

Bacterial Counts	Day 0	Day 14	Day 28	Day 42	Day 56	Day 70	Day 84
APC	4.89 ± 0.06 ^a^	5.00 ± 0.03 ^a^	4.91 ± 0.05 ^a^	4.96 ± 0.05 ^a^	5.07 ± 0.03 ^a^	5.08 ± 0.04 ^a^	5.09 ± 0.01 ^a^
PBC	5.30 ± 0.02 ^a^	4.93 ± 0.18 ^a^	5.01 ± 0.03 ^a^	5.13 ± 0.02 ^a^	5.13 ± 0.01 ^a^	5.17 ± 0.02 ^a^	5.12 ± 0.01 ^a^

Bacterial counts (Log CFU/g) were reported as means of four determinations ± standard deviation. Data with the same letter in the same row are not significantly different (*p* > 0.05).
